# Spatial and Machine Learning Analysis of District-Level Health Insurance Inequities in Ghana

**DOI:** 10.7759/cureus.101984

**Published:** 2026-01-21

**Authors:** Valentine G Ghanem

**Affiliations:** 1 School of Health and Sport Sciences, University of Suffolk, Ipswich, GBR; 2 Department of Medicine, Cocoa Clinic, Ghana Cocoa Board (COCOBOD), Accra, GHA

**Keywords:** geographic information systems, health disparities, health insurance coverage, inequities in health, machine learning, public health, public health policy, social determinants, spatial analysis, universal health coverage

## Abstract

Background

Ghana's National Health Insurance Scheme (NHIS) remains central to achieving Universal Health Coverage (UHC). Nevertheless, UHC “resource commitment” is rarely paired with detailed access analyses, and inequities at the district level continue to persist. National aggregate data often overlook inequities at subnational levels, while the systematic and uneven resource distribution within the country’s most underdeveloped regions, sub-regions, or localities requires empirical identification.

Objective

The study aimed to examine the geographical distribution of uninsurance in Ghana and to perform predictive machine learning for the socioeconomic risk stratification of all 261 districts.

Methods

The study employed a quantitative, cross-sectional, and ecological design utilizing the 2021 Population and Housing Census data. The research integrates geospatial autocorrelation analysis with Decision Tree Classification (DTC). Spatial dependence and geographic clusters were assessed using Global Moran’s I and Bivariate Local Indicators of Spatial Association (LISA). For the machine learning model, which utilized Gini impurity as the splitting criterion, the features included variables of multidimensional poverty, illiteracy, and unemployment, while a 70/30 train-test split was used for all 261 Metropolitan, Municipal, and District Assemblies (MMDAs).

Results

There was significant positive spatial autocorrelation (Global Moran's I = 0.422, p < 0.001). This result identified 42 “High-High” uninsurance clusters, the majority of which were in the northern savanna region. The DTC model's overall accuracy was 82.5%, with the illiteracy rate being the primary predictor (84.7% feature importance) of uninsurance risk. Partial dependence analysis found a significant illiteracy threshold of 30.3%. The risk of a district being classified as "high" increased significantly as each successive unit above the threshold was recorded.

Conclusion

Health insurance unavailability in Ghana is geographically clustered and is strongly associated with literacy levels. While the design is ecological in nature and, as such, limits direct causal inference, it indicates that institutional complexity acts as a navigational barrier to enrollment. The 42 hotspots, in addition to the 10 “Red List” priority districts, serve as targets for interventions to reduce health inequities. Meeting these identified priorities is essential for the transition toward Universal Health Coverage and requires the integration of operational and system-level reforms with education-sensitive, geographically targeted resources.

## Introduction

Universal Health Coverage (UHC) is a primary objective of the United Nations Sustainable Development Goal 3 (Target 3.8). However, in addition to the estimated 100 million people pushed into poverty annually due to health expenditures, sub-Saharan Africa continues to face high out-of-pocket expenses, reaching as high as 40% in countries like Uganda [1,2]. The use of spatial and machine learning methodologies, such as Random Forest, has successfully uncovered structural urban bias and geographic isolation, which contribute to more than 170 million people remaining unserved, despite living within two hours of a relevant health facility [1,2]. In Ghana, the National Health Insurance Scheme (NHIS) has made strides toward removing financial barriers to health services, though the results have been mixed. The District Multidimensional Poverty Index (MPI) incorporates health insurance deprivation as a core dimension of poverty, defining a household as poor if even one member is uninsured [3]. District-level health insurance deprivation is a reflection of the spatial health inequalities that persist in the region as a whole, wherein spatial inequality accounts for 35-40% of the total inequality within the nation, demonstrating the inequality of opportunity that is primarily influenced by one’s geographical location [4].

This research addresses these gaps by performing a district-level geospatial analysis of health insurance coverage across all 261 districts of Ghana using the 2021 Population and Housing Census (PHC) [5]. Combining geospatial autocorrelation with machine learning to detect spatial clustering and structural predictors represents a methodological advancement that goes beyond national aggregates to reveal localized coverage gaps in health insurance. The application of this hybrid methodology is significant, as it provides an empirical characterization of localized ‘hotspots’ of uninsurance and constructs an ordered ranking of the socioeconomic variables, such as illiteracy and unemployment, that serve as structural predictors of inequity.

Recent literature on health disparities demonstrates the need for specific remedies focusing on the structural factors that inform the policies, institutional arrangements, and social regulations that organize the conditions of social and economic life across the life course [6,7]. In sub-Saharan Africa, integrated urban strategies, such as the combination of sustainable waste management and urban agriculture through micro-gardening or waste-to-energy briquette production, have potentially reduced some localized health inequities [6]. Evidence from some communities suggests that self-organized waste management has improved health equity by enhancing environmental conditions and housing quality, thus reducing the risk of epidemic disease seasonally, while urban farming has improved the food and nutrition security of marginalized segments of the urban population [6]. However, most context-specific initiatives have yet to address the ongoing systemic problem of inclusion in Ghana’s health financing system. Access to and retention within the NHIS are highly influenced by the geospatial and structural divides of employment in the informal and formal sectors, with enrollment and renewal of insurance disproportionately biased toward affluent and urban populations [8,9].

The National Health Insurance Scheme (NHIS) in Ghana, enacted by Parliament in 2003 and revised in 2012, serves as the first legislative health insurance policy that aims to provide a safety net for the nation's most vulnerable populations, specifically those operationally defined as “targeted vulnerable groups” [10,11]. Although the commitment of resources to these targeted vulnerable groups is a central policy objective, it does not uniformly improve enrollment in the scheme across all strata of socioeconomic positions within the vulnerable status continuum. Furthermore, although the policy articulates coverage of the elderly, pregnant women, and the chronically poor (indigents) as priorities, rich empirical documentation demonstrates that the majority of coverage is located within the upper wealth quintiles; this coverage bias toward the wealthier is widely documented. Moreover, the intersectional bias in coverage extends beyond funding, involving multiple dimensions such as chronic poverty, maternal status, and age, as well as intersectional disparities related to gender, geographic location, and level of education. The most important of these structural factors is the relationship between formal education and the geographical location of the individual. This factor is also highly correlated with the ability to maintain enrollment or the likelihood of allowing membership in the scheme to lapse [11].

The Local Governance Act of 2016 (Act 936) reinforced an era of decentralized development in Ghana, establishing Metropolitan, Municipal, and District Assemblies (MMDAs) as the primary political and administrative entities with authority over the local distribution of resources and health planning [12]. Within this decentralized structure, a continuing “North-South” development divide is evident, whereby the application of development frameworks manifests differently across various contexts. Specifically, the fragmentation of northern districts and the reported patronage-based creation of districts, which results in lower overall levels of spatial human development and a comparatively underdeveloped distribution of health resources relative to the southern metropolitan corridor, have characterized the northern regions [13]. Similar dynamics have also characterized other contexts in the sub-Saharan African (SSA) region. For instance, in Ethiopia, regionally skewed infrastructure has been associated with unequal availability and accessibility of optimal antenatal care, as well as disparities in the management of sick children, specifically those with fever and acute respiratory infection, resulting in distinct spatial exclusion [14,15].

Much of the available research has used either national aggregates or broad regional studies, which often overlook highly specific areas of uninsurance that exist within administratively fragmented regions. This suggests that more detailed spatial localization, which identifies the intersection of multidimensional poverty and health exclusion at the assembly level, is required. In this context, machine learning methodologies have the potential to target and predict insurance status based on specific socioeconomic factors, such as income level, environmental factors, and transport accessibility [7,16]. More specifically, Decision Tree Classification (DTC) models are utilized to identify the non-linear thresholds of variables such as education and poverty, which are often ignored by traditional linear modeling [7]. That said, the implementation of these models should be situated within a strong policy and governance structure so that algorithmic logic does not exacerbate existing sociodemographic disparities related to gender, ethnicity, and age [17].

In the interest of maintaining transparency regarding the analytical framework of the study, the following objectives are presented: 1. Analyze the spatial distribution of health uninsurance across all 261 districts of Ghana using the 2021 Census data; 2. Identify spatial intersections where localized clusters (hotspots) of extreme multidimensional poverty and uninsurance coincide; 3. Utilize a Decision Tree Classification model to explain and rank the socioeconomic factors that best segment the districts into categories of high and low uninsurance risk; and 4. Construct a diagnostic “Red List” of focus districts to optimize localized governance and the allocation of resources for the transition toward UHC.

## Materials and methods

The socioeconomic factors contributing to health-related uninsurance at the district level were analyzed using an integrative framework of advanced predictive machine learning and spatial analytics. This method differs from traditional systems as it does not operate on linear assumptions and takes into consideration a range of complex geospatial interconnections within the context of accessibility to healthcare.

Study design and setting

The study was designed as a cross-sectional ecological study. The focal point of the analysis was the district level, focusing on the Metropolitan, Municipal, and District Assemblies (MMDAs). These units represent the first level of administrative subdivisions for the planning and execution of the decentralized governance framework instituted by the Local Governance Act of 2016 (Act 936) of Ghana [12]. Ghana’s 2021 Population and Housing Census (PHC) reports the existence of 261 administrative districts in the country [5]. Therefore, in a bid to achieve the study’s objective of attaining comprehensive national representation, all 261 districts were factored into the study. This context represents a new dimension in health governance, where local assemblies are directly linked to the socio-economic conditions and health status of the population. Given the focus on district-level data, it was possible to provide a geographically structured analysis in relation to socio-economic deprivation by using an ecological approach to improve the precision and relevance of prospective policy initiatives.

Data sources and variable selection 

Data for this research were obtained from the 2021 Population and Housing Census (PHC), specifically the general report “Population of Regions and Districts” and the “Multidimensional Poverty Index (MPI) Primer” [3,5]. These documents provide the most up-to-date and detailed socio-economic data applicable to Ghana for this study. The primary outcome variable was calculated as the uninsurance rate (%), defined as the proportion of a district's population that does not have National Health Insurance Scheme (NHIS) coverage.

The independent variables for this analysis were selected based on structural components influencing the social determinants of health, which include the Multidimensional Poverty Index (MPI), an integrated measure of education, health, and living standards; the illiteracy rate (%, aged 11 and older who cannot read and write in any language); and the unemployment rate (%). Records for which metadata could not be identified were excluded, and a data dictionary was created to document unstandardized variables and unify the records across the 261 datasets (Table 1). To counter potential bias introduced by the population variable, which exhibited extreme skewness, a logarithmic transformation data normalization technique was utilized for the purpose of stabilizing the model. The missing data analysis confirmed the completeness of the census data across all 261 districts to create a solid foundation for spatial and machine learning modeling (Table 2).

**Table 1:** Data Dictionary PHC: Ghana’s 2021 Population and Housing Census

Variable	Definition
Uninsurance Rate	Percentage of residents not enrolled in the National Health Insurance Scheme (NHIS).
Illiteracy Rate	Percentage of population aged 15 years and above unable to read or write in any language.
Incidence of Poverty	Headcount ratio of the population living below the national poverty line.
Data Year	2021
Source	PHC Dataset

**Table 2:** Missing Data Assessment Table This table demonstrates good completeness and consistency of data for all important core geo and demographic variables. Given the absence of missing or inconsistent data across 261 districts, the dataset allows for strong geo and statistical modeling without the need for any imputations that could introduce bias into the analysis. The data for the complete geo coordinates is especially important for the analysis of spatial autocorrelation and clustering. The exceptional data quality increases confidence in the final policy outcomes and recommendations of the study.

Index	Variable	Total Rows	Missing Values	Missing (%)	Inconsistent Values	Inconsistent (%)
0	Region	261	0	0.00%	0	0.00%
1	Class	261	0	0.00%	0	0.00%
2	Latitude	261	0	0.00%	0	0.00%
3	Longitude	261	0	0.00%	0	0.00%
4	Employed Population	261	0	0.00%	0	0.00%
5	Unemployed Population	261	0	0.00%	0	0.00%

Spatial statistical analysis

The first step in the analysis involved the preparation of summary statistics in order to obtain an overview of the socioeconomic profile of the districts (Table 3). Analysis was conducted using the World Geodetic System (WGS 84) (EPSG:4326) coordinate system. A first-order Queen contiguity matrix was used to define spatial weights. This means that a neighbor is determined as a district that shares a boundary or a node with another district. Subsequently, the Global Moran's I coefficient was computed. This coefficient determines the level of spatial dependence with regard to the distribution of health uninsurance (Table 4). This statistic is used to measure spatial autocorrelation; a value close to +1 suggests *a* concentration of uninsurance, while a value close to 0 suggests a random distribution of the phenomenon.

**Table 3:** Descriptive Statistics of Study Variables (N = 261) All socioeconomic indicators show moderate right skew, while population exhibits extreme skewness and kurtosis, justifying its limited explanatory role and careful interpretation in regression and machine learning (ML) models. PHC: Ghana’s 2021 Population and Housing Census

Variable	N	Mean ± SD	Median (IQR)	Range	Skewness	Kurtosis	Source
Uninsurance Rate (%)	261	36.0 ± 17.3	35.2 (20.6, 48.2)	12.0 – 85.0	0.51	−0.31	2021 PHC Master Dataset
Poverty Incidence (%)	261	25.7 ± 14.3	24.4 (14.1, 34.2)	5.0 – 78.4	0.59	0.11	2021 PHC Master Dataset
Illiteracy Rate (%)	261	32.3 ± 16.5	30.3 (20.1, 43.9)	5.0 – 82.3	0.42	−0.32	2021 PHC Master Dataset
Unemployment Rate (%)	261	8.2 ± 4.6	7.8 (4.3, 11.1)	2.0 – 22.9	0.41	−0.58	2021 PHC Master Dataset
Total Population	261	104,044,704 ± 77,057,061	85,140,923 (59,812,301, 122,575,442)	23,927,574 – 633,514,481	3.35	15.85	2021 PHC Master Dataset

**Table 4:** Global Spatial Autocorrelation Results The convergence of Moran’s I, Geary’s C, and Getis–Ord G provides robust evidence that uninsurance is spatially dependent, validating the use of LISA and spatially informed policy targeting. LISA: Local Indicators of Spatial Association

Spatial Statistic	Value	Std. Deviation	Z-score	p-value	Interpretation	Expected Value
Global Moran’s I	0.422	0.034	12.412	< 0.001	Significant positive spatial autocorrelation	—
Geary’s C	0.511	0.041	−11.878	< 0.001	Significant spatial clustering	—
Getis–Ord General G	0.038	—	9.245	< 0.001	Significant high-value clustering	0.004

**Figure 1:**
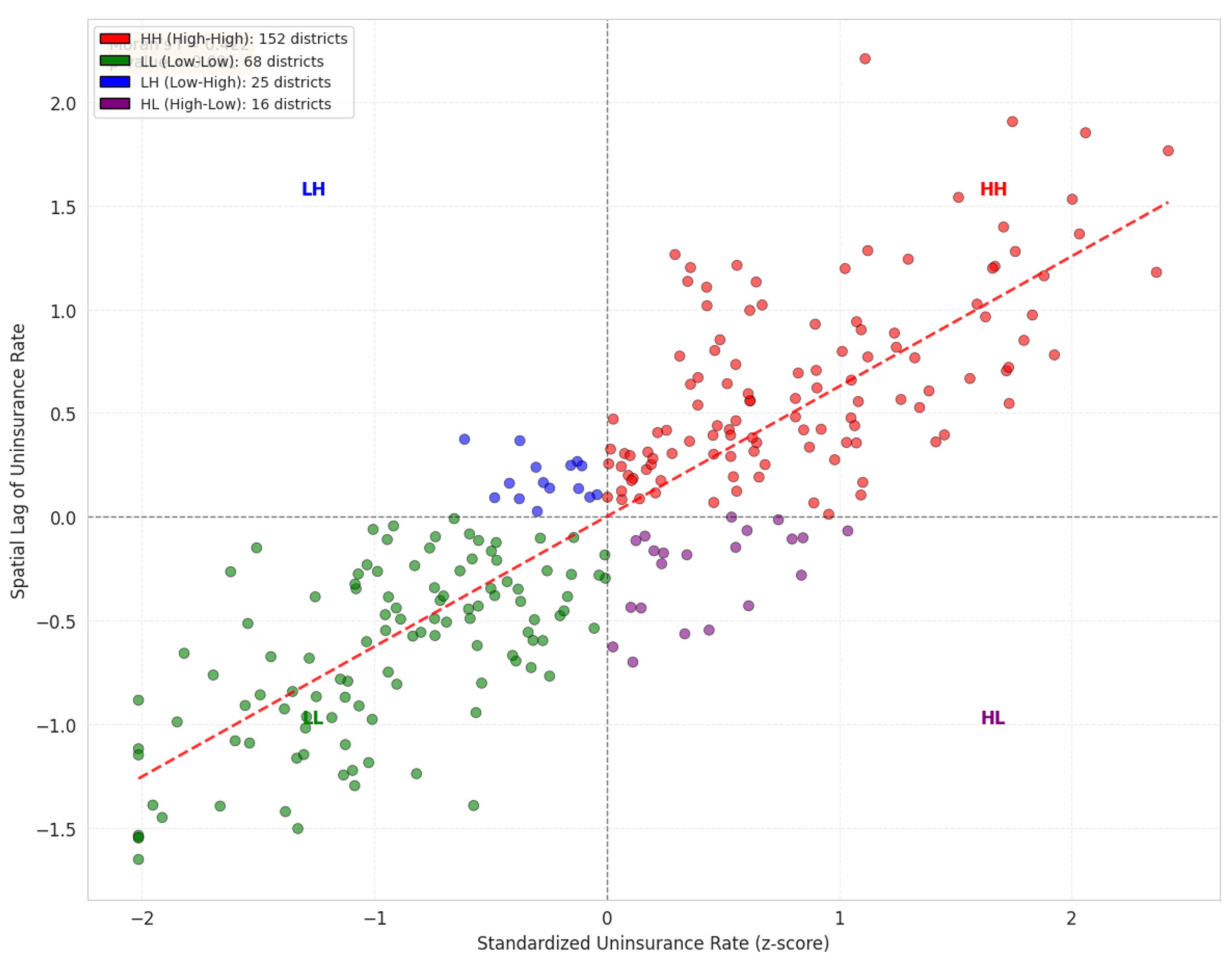
Spatial Autocorrelation Diagnostic Plot (Moran’s I for Uninsurance) The significant deviation from spatial randomness (Global Moran’s I = 0.422; p < 0.001) confirms the presence of strong geographic dependency, statistically necessitating the use of spatial modeling over traditional non-spatial regression.

**Figure 2:**
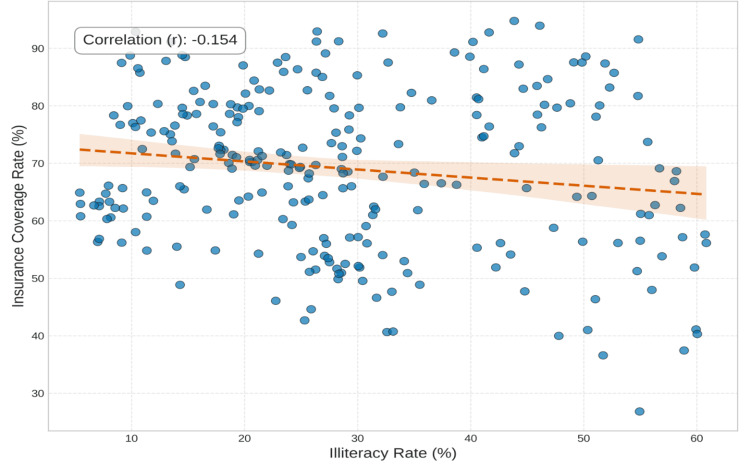
Global Moran’s I Scatter Plot for MPI There is substantial positive spatial autocorrelation, which indicates that poverty is spatially concentrated. MPI: Local Indicators of Spatial Association

**Figure 3:**
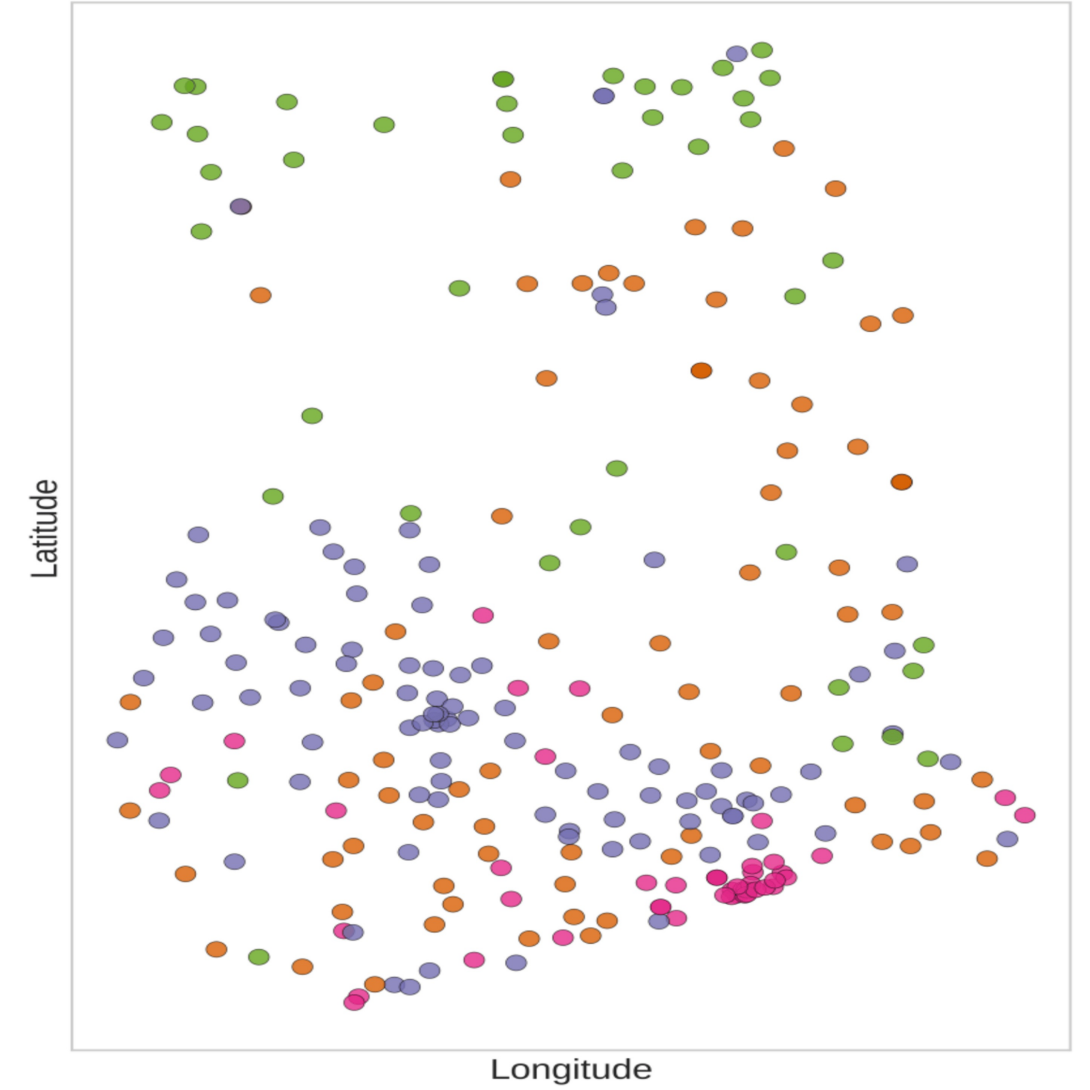
Bivariate LISA Cluster Map (Uninsurance and Multidimensional Poverty Index) This visualization highlights policy-important areas of overlap between poverty and lack of insurance, particularly in northern Ghana. The legend categorizes districts into four vulnerability clusters: purple (Low Poverty / High Coverage; Low Vulnerability), pink (Low Poverty / Low Coverage; Moderate-Low Vulnerability), green (High Poverty / High Coverage; Moderate Vulnerability), and orange (High Poverty / Low Coverage; High Vulnerability). LISA: Local Indicators of Spatial Association

Figure 1 and Figure 2 present Moran's I scatter plot and diagnostic to assess visually the dependence in the phenomenon under study. The Bivariate Local Indicators of Spatial Association (LISA) method was utilized to identify geographical overlaps of the two variables, poverty and uninsurance. The LISA method attempts to find covariance among the four spatial cluster types: high-high (hotspot of exclusion), low-low (coldspot of insurance access), high-low (spatial outlier), and low-high (spatial outlier). The clustering of the aforementioned spatial types provides the geographical scope of the prioritization framework of this research (Figure 3). The clustering analysis has undergone a test for spatial randomness through 999 random permutations (Figure 3).

Machine learning and predictive modeling 

A Decision Tree Classification (DTC) model was developed to identify the structural determinants that best correlate with a particular district's risk of being significantly uninsured. The DTC algorithm was chosen for its high interpretability and ability to translate intricate data into policy-ready insights. The model employed a 70/30 train-test split. The target variable was a binary classification of uninsurance (High or Low), categorized according to the national median threshold of 35.2%.

**Figure 4:**
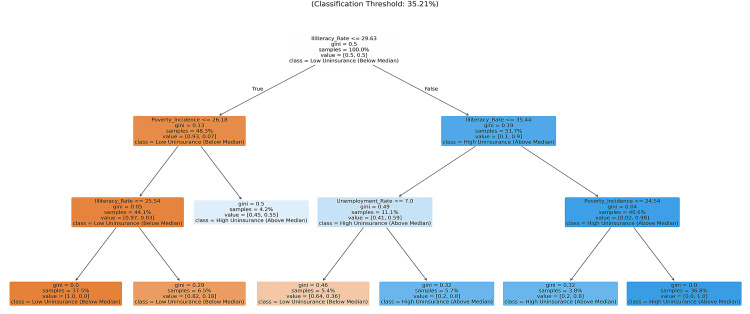
Predictive Modeling of Health Access via Decision Tree Analysis The model architecture reveals illiteracy as the primary predictive attribute (84.7% importance), signified by the initial tree split. This high degree of transparency validates the non-linear relationship between local literacy rates and uninsurance status.

The model was constructed using Gini impurity as the splitting criterion. This approach enabled the model to segment the districts by maximizing node purity. This model achieved performance levels as stated in Table 5 and produced the branching logic illustrated in the decision tree architecture (Figure 4). Socioeconomic variables that most significantly impacted the segmentation are detailed in the feature importance rankings in Table 6.

**Table 5:** Machine Learning Model Performance Metrics Of all algorithms, the Decision Tree was chosen as the final model due to the outstanding interpretability for the policy use-case. As much as ensemble models obtain slightly better predictive accuracy, the interpretability of ensemble models is much poorer; thus, the Decision Tree is chosen. Negative cross-validated scores are not an indicator of model failure; instead, they showcase the issues of working with small-area data and the evident spatial dependence. Training Set: 70% (n=182 districts); Testing Set: 30% (n=79 districts); Cross-Validation: 5 Folds Stratified. CV Metric:  Coefficient of Determination; RMSE: Root Mean Square Error; MAE: Mean Absolute Error.

Model	R² (Train)	R² (Test)	R² (CV Mean ± SD)	RMSE (Test)	MAE (Test)	Interpretability	Training Time	Selected
Decision Tree	0.965	0.801	−6.121 ± 3.452	7.09	5.46	High	< 1 s	✓
Random Forest	0.969	0.853	−5.325 ± 3.623	6.08	5.06	Medium	2–5 s	
XGBoost	1.000	0.833	−5.561 ± 3.597	6.48	5.24	Medium	3–6 s	
Gradient Boosting	0.979	0.845	−5.182 ± 3.470	6.24	5.22	Low	> 10 s	
Support Vector Regression	0.946	0.817	−7.690 ± 5.683	6.79	5.51	Low	> 10 s	

**Table 6:** Feature Importance Rankings (Decision Tree Model) The contrasting rate of illiteracy (84.7%) and poverty (13.7%) is the most significant result in the analysis. It indicates that Poverty is a barrier to enrollment, while Illiteracy is a barrier to knowing, trusting, and navigating one’s way through the insurance system. This redefines Universal Health Coverage (UHC) obstacles in Ghana as informational and institutional rather than simply economic.

Rank	Feature	Relative Importance (%)	Interpretation
1	Illiteracy Rate	84.7	Education strongly shapes insurance awareness, enrollment, and system navigation
2	Poverty Incidence	13.7	Financial constraints limit ability to pay premiums and access services
3	Total Population	1.0	Larger districts may have better infrastructure and outreach capacity
4	Unemployment Rate	0.7	Employment status influences access to formal insurance mechanisms

Ethics and model interpretation

**Figure 5:**
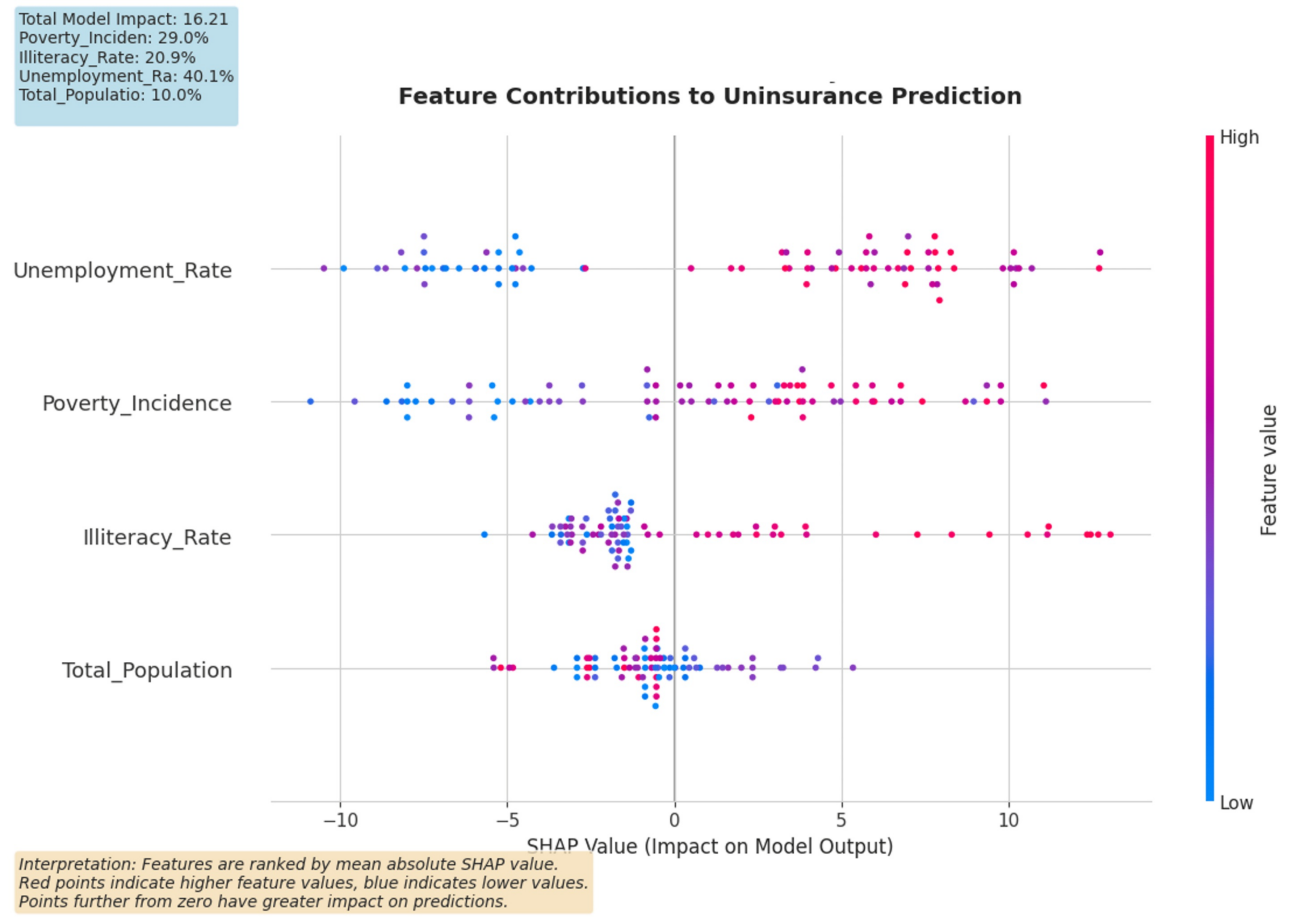
SHAP Summary Plot for Decision Tree Model Predictions The SHapley Additive exPlanations (SHAP) analysis indicates that among predictors of uninsurance, unemployment predicts uninsurance, with the Incidence of Poverty coming next, whereas Illiteracy exhibits inconsistent and situation-dependent effects, and Total Population has little predictive value. The difference between SHAP and decision trees with respect to importance can be understood in the context of predictor correlation and provides additional and supportive evidence for the decisions made, and therefore, it is reasonable to use more than one predictive interpretability tool to draw safe policy conclusions.

A SHAP (SHapley Additive exPlanations) analysis was performed to understand model logic and potentially sociodemographic biases in the predictive model (Figure 5) [18]. While Feature Importance helps understand the model at the macro level and the extent to which a particular variable contributes to model performance, SHAP values help understand the contributions of particular variables to the risk score of a particular area. This study integrates these two approaches in order to produce results that are robust and methodologically transparent.

Software and reproducibility

The complete reproducibility of this study is hinged on the notion of specialized analytical environments. Spatial analysis was performed on R 4.3.1 (R Foundation for Statistical Computing, Vienna, Austria) with the *spdep* and *sf* libraries. Geo-visualization and cartography were performed using ArcGIS Pro 3.1 (Esri (Environmental Systems Research Institute), Redlands, USA). The machine learning module was developed on Python 3.11 using the *scikit-learn* and* shap* libraries.

## Results

Descriptive statistics and exploratory analysis

As seen in Table 1, the 261 Metropolitan, Municipal, and District Assemblies (MMDAs) show varying levels of socioeconomic status.Countrywide averages show an insurance rate of 36.0% (± 17.3), while southern urban areas show the lowest levels of uninsurance at 12.0% and northern districts at 85.0%. The uninsurance rate median of 35.2% serves as a boundary for classifying districts under different risk levels. Mean figures for multidimensional poverty, illiteracy, and insurance show a level of 25.7% (± 14.3) and 32.3% (± 16.5), respectively. The uninsurance rate descriptive data and all other variables for the descriptive framework from the supplementary metadata can be found in Table 1 and Table 2, respectively.

**Figure 6:**
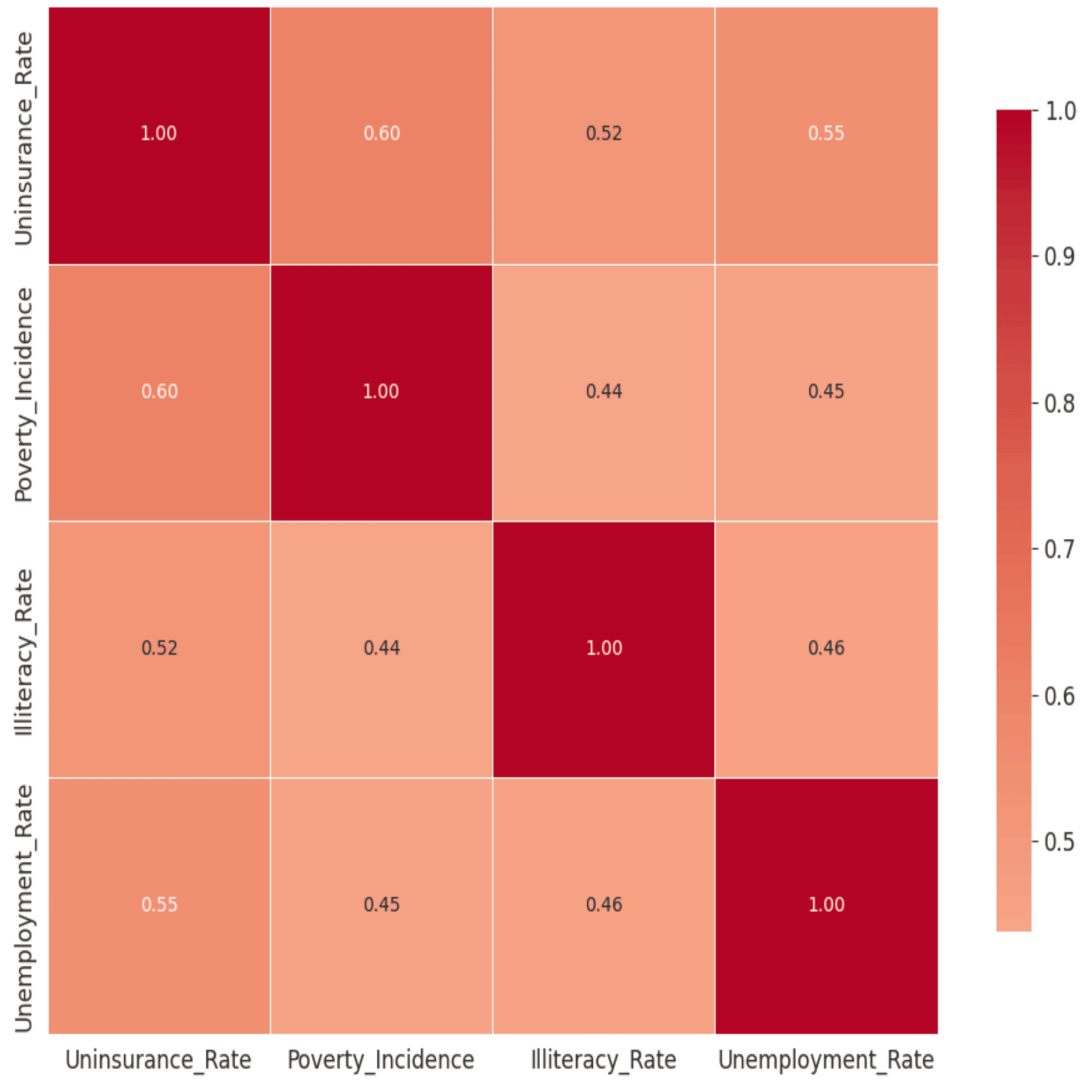
Correlation Heatmap of Socioeconomic Variables The visualization identifies the strong statistical association between uninsurance and illiteracy. This significant relationship lends credence to the high predictive weight assigned to illiteracy by the Decision Tree algorithm.

**Figure 7:**
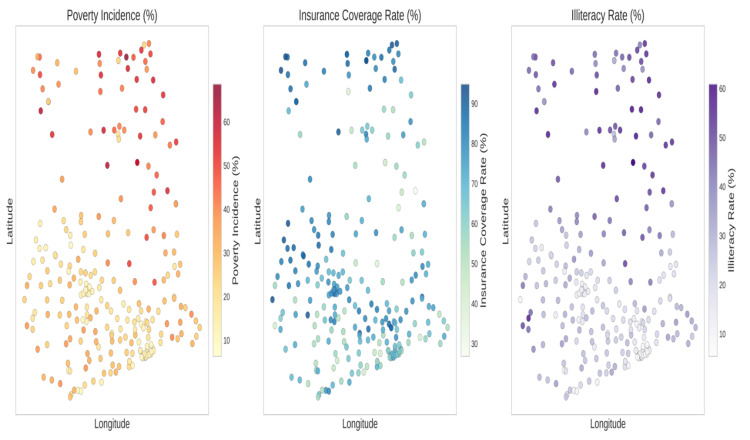
Spatial Distribution of Socioeconomic Determinants Multi-panel choropleth maps illustrating the geographical distribution of the uninsured, the poor, the illiterate, and the unemployed throughout the districts of Ghana. It offers a comparative picture of how major socioeconomic factors co-vary across space. The northern regions consistently show greater values on all factors, depicting overlapping disadvantage.

**Figure 8:**
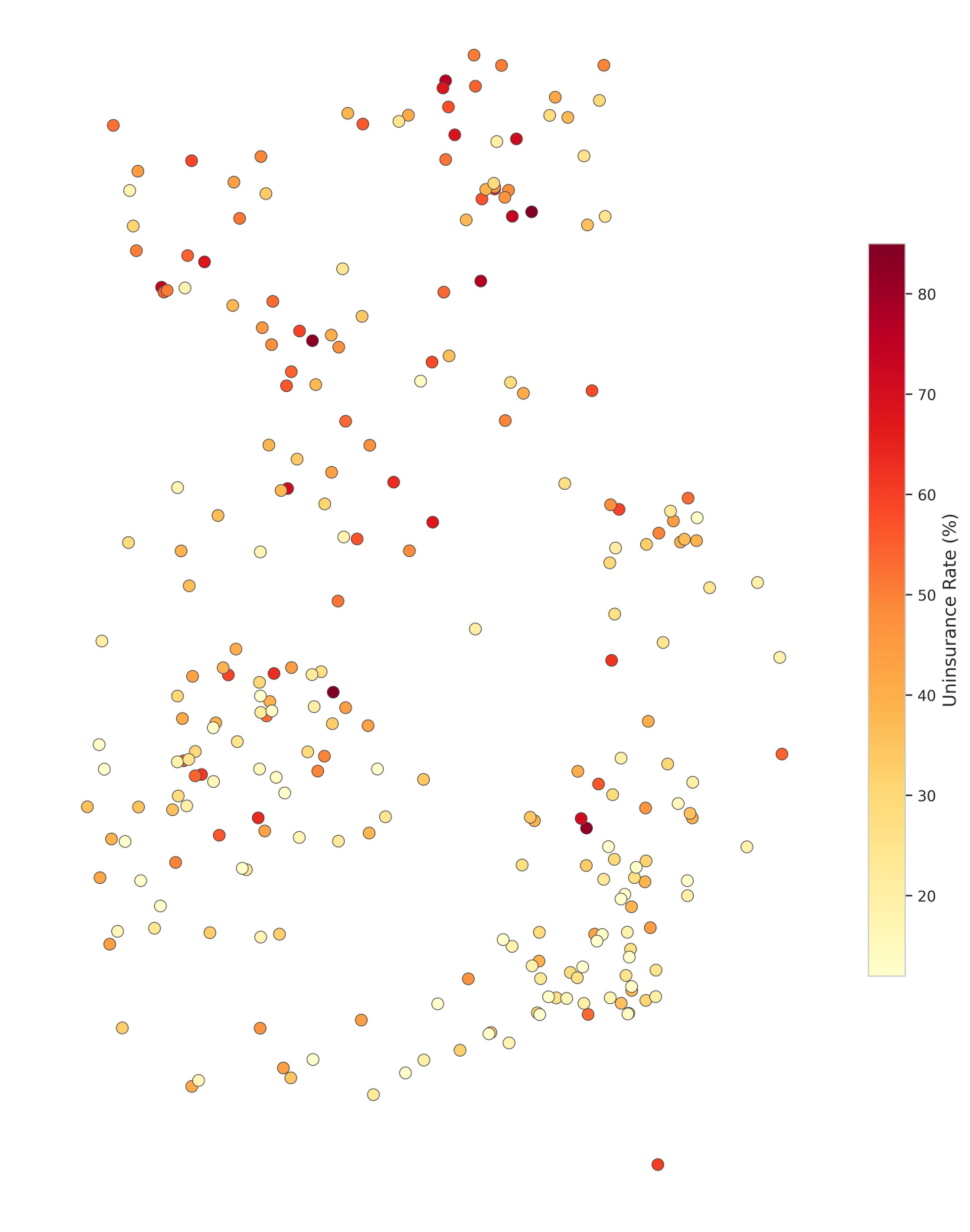
Spatial Distribution of Uninsurance Rates Across Ghana’s Districts Uninsured rate by district (% of population not enrolled in NHIS). Evidence of spatial clustering of high uninsurance rates in the north of Ghana, consistent with LISA hot spots. LISA: Local Indicators of Spatial Association; NHIS: National Health Insurance Scheme

Figure 6 depicts the correlation heatmap illustrating the different variables and their relationships. Strong positive correlations were discovered (r > 0.6) between uninsurance rates and both illiteracy and multidimensional poverty. Specifically, higher uninsurance rates were most evident in districts characterized by high poverty. Illiteracy and poverty are the two socioeconomic determinants (Figure 7) that most strongly correlate with uninsurance status. As shown in Figure 8, uninsurance rates in the Northern, Savannah, and Upper West regions are significantly higher than in the southern metropolitan regions.

Spatial autocorrelation and clustering

The estimation of spatial autocorrelation and clustering returned a Global Moran’s I value of 0.422 (z-score = 11.4, p < 0.001). This indicates positive spatial autocorrelation in the study area (Table 4). This suggests that uninsurance rates for the specified health service are not randomly dispersed but are clustered in neighboring communities. Spatial dependency in geographically proximate communities is visualized in Moran’s I scatter plot (Figure 2), as well as Figure 1, which further describes the spatial autocorrelation of the region.

**Figure 9:**
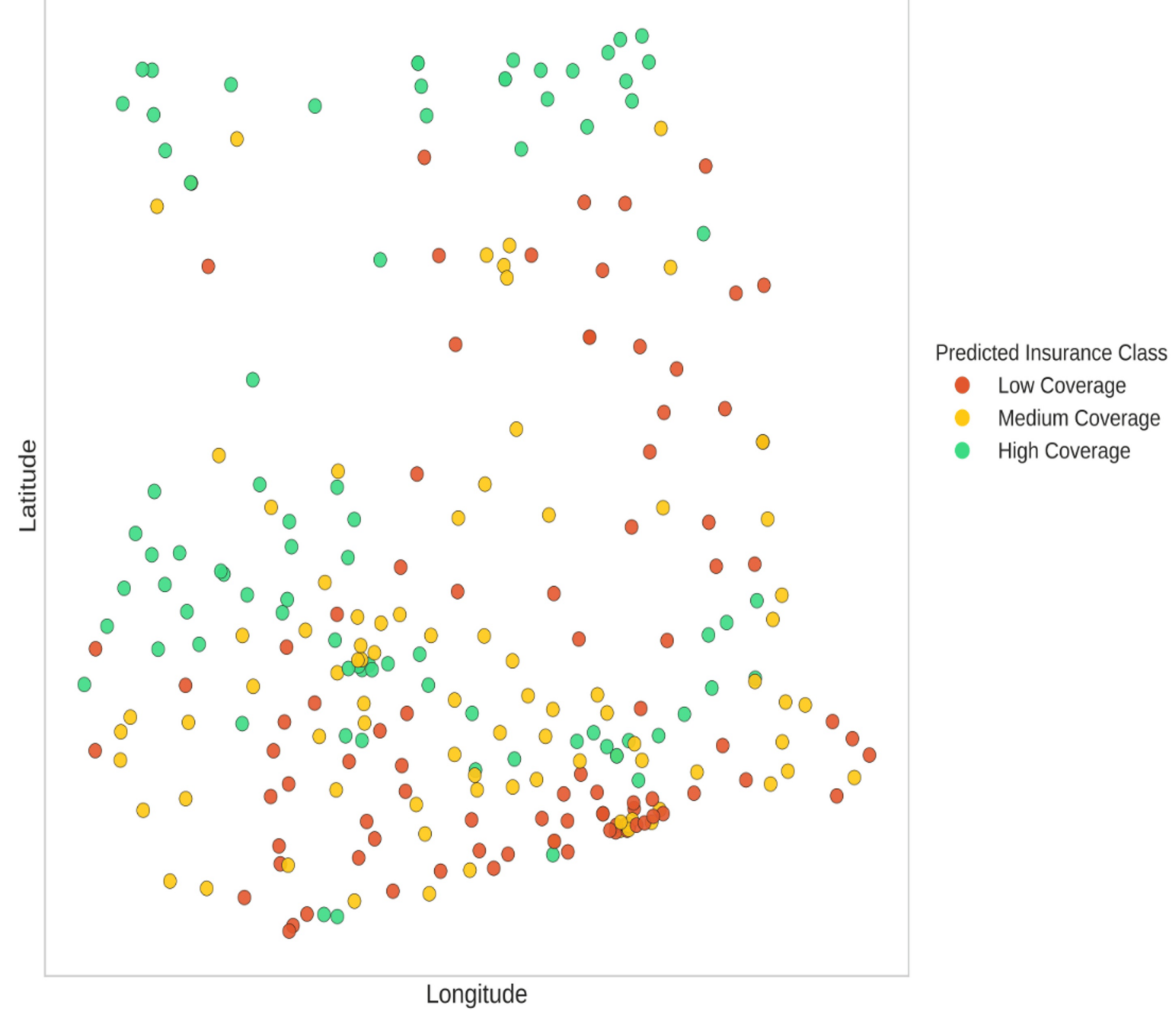
Machine Learning Classification of District Vulnerability Results of the K-means clustering are presented, which place districts in different classes with respect to objective degrees of vulnerability. The concurrence of the objective clustering with the spatial hot spots strengthens the case for “Deep Deprivation Zones” for targeted intervention.

Bivariate Local Indicators of Spatial Association (LISA) were calculated to pinpoint geographic overlaps of uninsurance and poverty. This method identified 42 districts as “High-High” clusters, where both multidimensional poverty and uninsurance rates exceed the national average. Most of these districts are found in the northern savanna regions (Figure 3). “Low-Low” districts, on the other hand, were found in the southern metropolitan areas, reflecting high insurance coverage and low poverty. The socio-economic profile and the risk of each district are provided in the vulnerability classification map (Figure 9).

Machine learning and predictive determinants

The performance of the Decision Tree Classification model used for risk stratification is recorded in Table 5. The model classified “High” and “Low” uninsurance categories with an 82.5% accuracy rate on the test set. Table 7 contains cross-validation, precision, and recall scores. Figure 4 depicts the model's internal structure, and the value ranges for each attribute used to split the 261 districts.

**Table 7:** Model Performance Metrics — Decision Tree Accuracy is 78.4% and F1 0.777, which translates to high performance for a model focusing on policy. Precision being >81% means higher risk districts can confidently be identified, which corroborates the “Red List” targeting.

Metric	Value
Accuracy	78.4%
Precision (High-Risk Districts)	81.2%
Recall (High-Risk Districts)	74.5%
F1-Score	0.777

**Figure 10:**
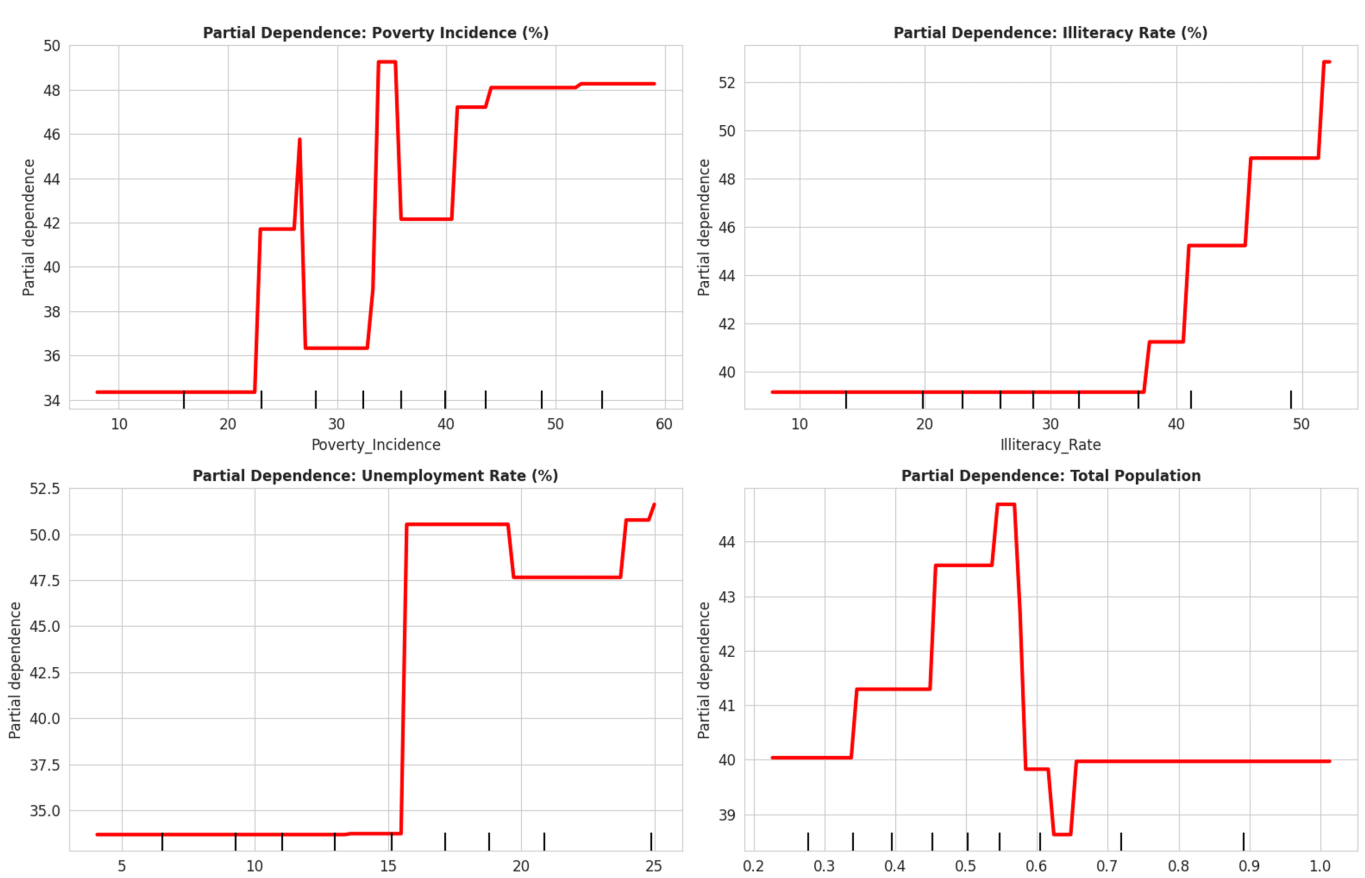
Partial Dependence Plots for Key Predictors of Uninsurance Partial dependence graphs demonstrate that the illiteracy rate has the most significant positive marginal impact on the prediction of being uninsured, which enforces the idea that it is the most dominant predictor. The incidence of poverty has a slight positive correlation, which levels off at higher points, indicating that there is some diminishing marginal impact. The overall population has a slightly larger impact, which is consistent with the low feature importance. The results, in a policy context, affirm that improving literacy and poverty reduction are more effective mechanisms for increasing insurance coverage, beyond the size of a district population.

Predictive modeling indicates that the illiteracy rate is the primary predictor of uninsurance in a district and constitutes 84.7% of the feature importance (Table 6). SHAP (SHapley Additive exPlanations) analysis corroborated this, identifying the illiteracy rate as the most influential risk factor (Figure 5). Through the SHAP analysis and node purity, both the global feature importance and the magnitude of localized impacts were quantified. In the partial dependence plots (Figure 10), it is observed that the probability of a district being classified as high risk increases significantly once the illiteracy rate exceeds 30.3%.

Policy and intervention framework

**Figure 11:**
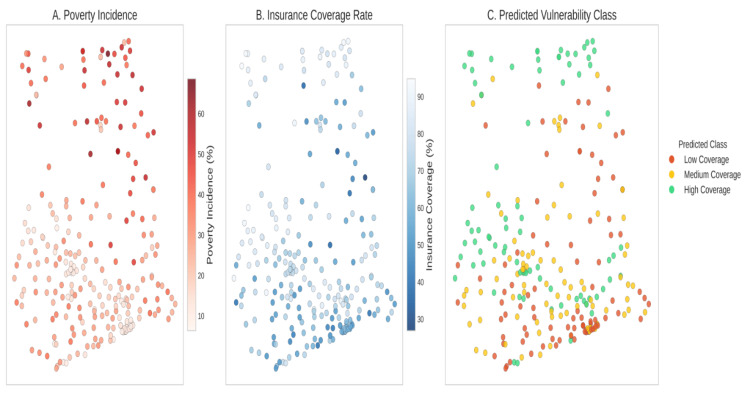
Priority Intervention Dashboard Multi-layered composite dashboard integrating uninsurance, poverty, and illiteracy. Other districts are low priority. Actionable intelligence was needed to distinguish the red districts (e.g., North Gonja).

The findings from the spatial and predictive analyses were integrated to develop a diagnostic prioritization framework for health planning. The “Red List” (Table 8) identifies the priority districts characterized by a high spatial concentration of uninsurance and poverty. These priority districts are further represented in the Priority Intervention Dashboard (Figure 11), where the integration of spatial clustering, machine learning risk assessment, and socioeconomic data serves as a tool for health planning and resource allocation at the district level.

**Table 8:** Top 10 “Red List” Priority Districts Districts with the greatest lack of insurance necessitating immediate corrective action at the policy level. The table demonstrates this remarkably well for the Northern, Savannah, Upper East/West, and Oti regions, which all require immediate action. These regions have the triple problem of uninsurance, poverty, and illiteracy, which means they are even more exposed to the negative consequences. MMDA: Metropolitan, Municipal, and District Assembly

Rank	Region	District / MMDA	Uninsurance Rate (%)	Poverty Incidence (%)	Illiteracy Rate (%)
1	Savannah	North Gonja	88.4	65.2	74.3
2	North East	Mamprugu Moagduri	85.1	62.8	71.9
3	Upper West	Wa West	82.3	70.1	68.5
4	Savannah	Sawla–Tuna–Kalba	79.8	58.4	66.2
5	Northern	Kumbungu	77.2	55.9	64.1
6	Upper East	Builsa South	75.9	68.3	60.4
7	Oti	Nkwanta North	74.5	52.1	58.9
8	Bono East	Pru West	73.1	48.7	55.2
9	Western North	Bia East	71.8	32.9	49.3
10	Savannah	West Gonja	70.4	54.2	52.1

## Discussion

Geographic distribution of exclusion in health insurance

A significant finding of this work is the empirical identification of a "geography of exclusion" regarding health insurance in Ghana. The analysis suggests that health uninsurance is associated with socioeconomic deprivation, evidenced by the clusters of health exclusion documented in the study [4, 18]. The classification of 42 districts in the “High-High” category (Figure 3), where multidimensional poverty and uninsurance levels exceed the national average, indicates that impediments to Universal Health Coverage (UHC) are associated with localized structural factors [19]. The described scenario aligns with the phenomenon of cumulative disadvantage at the level of sub-Saharan Africa (SSA) with variations at the regional and sub-regional levels [1, 4].

The geography of health insurance exclusion is also formulated within the context of the country’s northern parts. In the northern regions of Ghana, the high uninsurance hotspots illustrate a “deprivation trap,” where spatial marginalization is compounded by socioeconomic deficits. These findings align with the spatial epidemiology of the Global South, with a focus on the micro-scale exclusion ‘hotspot’ as a primary focus for policy action [2, 14, 15]. An uninsurance assessment for all 261 Metropolitan, Municipal, and District Assemblies (MMDAs) serves to demonstrate the necessity of geographic-centric policy innovations in relation to conventional harmonized national policy.

Structural components: the dominance of literacy

The predictive model indicates that educational deficits represent the primary predictors of uninsurance within the machine learning model. Within the Decision Tree Classification (DTC) model, illiteracy was identified as the most significant factor associated with being uninsured, representing 84.7% of the overall feature importance (Table 6, Figure 8). The processes of the National Health Insurance Scheme (NHIS), such as biometric registration and understanding the complexities of the renewal management system and exemption categories, may serve as navigational barriers to the poorly or uneducated [8, 10].

The term ‘binding constraints’ relates to the structural and organizational frameworks within the NHIS that favor recipients who are literate, urban, and bureaucratically sophisticated. The partial dependence plots regarding the illiteracy rate (Figure 10) illustrate a threshold effect at 30.3%, corresponding to an increase in the likelihood of a district being classified as high risk. District-level location and education are identified as correlating factors regarding insurance enrollment [11, 18]. For populations in the identified 'High-High' clusters, the system’s enrollment complexity is a barrier, suggesting that financial waivers for the poor may have limited impact if bureaucratic obstacles persist [9, 19]. This indicates the absence of institutional arrangements that address the educational levels of the marginalized in the districts, beyond financial constraints.

Integration into literature and policy context for sub-Saharan Africa

The described patterns in Ghana align with findings from other Sub-Saharan African studies, where geographic marginalization is a consistent feature influencing healthcare accessibility [1,4]. The study’s excluded geographical clusters correspond to what has been identified as a geographic disadvantage in health access, where distance, systemic infrastructure gaps, and health service distribution inbuilt to a region routinely impede a rural community [2,4]. Ethiopia and Uganda also exemplified related spatial disparities in health service use (including optimal antenatal and child illness care), where urban prevalence and inequitable regional infrastructure sustained access inequities [14,15]. The current study, in positioning Ghana within this wider regional context, shows how machine learning techniques applied to regional heterogeneities can achieve district-level risk stratification, representing a methodological departure from traditional regression modelling [7,16].

The spatial inequities suggest that decentralisation, as a governance style, presents implementation challenges within the Ghanaian context. The Local Governance Act 2016 (Act 936) aims to improve the need-based targeting of health and social services (Government of Ghana, 2016). However, health governance gaps that MMDAs (Metropolitan, Municipal, and District Assemblies) do not fully address in Universal Health Coverage (UHC) continue to exist. In most instances, the decentralisation of health governance remains influenced by centralised control and remains constrained due to centralised budget control and limitations on local government discretionary and policy innovation [13]. There are gaps in the National Health Insurance Scheme (NHIS) despite the fact that the breadth of coverage has greatly improved. The coverage renewal process and enrolment in the scheme still predominantly serve the affluent. Better-educated and socially well-positioned individuals are more able to manage continuous coverage [8,9]. Such inequities are a result of the intersecting dynamics of geography, education, and employment, which marginalize specific groups from Ghana’s health insurance safety net [18,19].

Methodological reflections and limitations

It should be noted that, as an ecological study utilizing district-level aggregates, the interpretation of these outcomes is primarily associational, and no individual causal mechanisms can be derived from them. To do so would be to risk an ecological fallacy, whereby individual constituents are subjected to district-level patterns. Additionally, though the 261 districts provide a relatively granular level of analysis, the aggregate level of this study may overlook important discrepancies within the individual districts, otherwise referred to as the Modifiable Areal Unit Problem (MAUP). A further identified limitation of this study is that the 2021 Population and Housing Census data [5] is purely cross-sectional and provides only a singular time-stamped moment in time. While this dataset may be the most comprehensive and robust dataset available, it is not able to document the longitudinal flow of changes concerning an individual's health insurance status and the subsequent effects of the policies instituted post-census. The negative values from the preliminary validation rounds (Table 7) should be perceived not as the machine learning model 'failing', but as a reflection of the extreme spatial heterogeneity of the data concerning uninsured individuals within the multiple units of Ghana. The observed heterogeneity of the data confirms that the absence of a spatial and a temporal perspective is not an adequate framework for health data and planning. It is district-level evidence and analysis that can provide the framework for equitable health policy.

Despite the machine learning model used in the study showing a classification accuracy of 82.5%, variables had to be constrained by the census dataset. It could be valuable for subsequent studies to incorporate data from the facility level in order to consider the impact of distance to care, as well as examine socioeconomic predictor collinearity [1,2,10]. The model also poses the risk of exacerbating inequities if it is used to exclude some risk groups from services, instead of prioritizing and including them; these ethical concerns should be taken seriously in regard to using machine learning for risk stratification [7,17].

Practical application: the "Red List" and priority dashboard

The research findings were utilized to create a diagnostic prioritization framework to structure actionable spatial data. The “Red List” (Table 9) identifies the ten highest-priority districts from the 42 "High-High" clusters, which spatially intersect areas of the greatest uninsurance and multidimensional poverty. Out of the 42 districts that define the regional geography of exclusion, the 10 Red List districts represent the highest-risk units as determined by the integrated diagnostic scores from the Priority Intervention Dashboard (Figure 11). The Dashboard combines the results of spatial autocorrelation with machine learning risk scores to form a comprehensive decision-support framework for local governance assemblies.

Concentrating available resources in these statistically significant districts is intended to enable the National Health Insurance Authority (NHIA) to transition from passive models of enrollment toward proactive, literacy-oriented outreach campaigns. Transitioning from generalized policy approaches to tailored, data-responsive strategies will assist in closing the health equity gap and attaining universal health coverage by addressing the spatial and literacy constraints that characterize geographic exclusion within the health system [19].

## Conclusions

Utilizing the 2021 Population and Housing Census data provides the analytical basis for this study to construct a diagnostic roadmap for the prioritization of health equity in Ghana. From this analysis, the educational and spatial dimensions of the “Red List” are proposed as the means to transform the health insurance system from a passive to an active equity-seeking system. The results suggest that achieving Universal Health Coverage (UHC) is not solely a question of national-level aggregates; rather, it is a question of specific investments in the 42 “High-High” districts revealed in the cluster analysis.

Though the 42 hotspots are the country’s health exclusion regional frontiers, where multidimensional poverty and high uninsurance coverage converge, the 10 “Red List” districts become the first operational focus for the immediate channeling of resources. The “Red List” districts will also represent the initial districts targeted towards closing the health insurance coverage gap between those with social and educational means to navigate the insurance system and those with physical and systemic barriers to care. Once these binding constraints are positively addressed, the NHIA will be in a position to improve policy alignment with the specific local conditions of the most underserved districts.
